# Correction: Scharf et al. Oxford Nanopore Technology-Based Identification of an *Acanthamoeba castellanii* Endosymbiosis in Microbial Keratitis. *Microorganisms* 2024, *12*, 2292

**DOI:** 10.3390/microorganisms13071497

**Published:** 2025-06-27

**Authors:** Sebastian Alexander Scharf, Lennart Friedrichs, Robert Bock, Maria Borrelli, Colin MacKenzie, Klaus Pfeffer, Birgit Henrich

**Affiliations:** 1 Institute of Medical Microbiology and Hospital Hygiene, Medical Faculty, Heinrich Heine University Düsseldorf, Moorenstrasse 5, 40225 Duesseldorf, Germany; lennart.friedrichs@med.uni-duesseldorf.de (L.F.); colin.mackenzie@med.uni-duesseldorf.de (C.M.); klaus.pfeffer@hhu.de (K.P.); 2 Department of Ophthalmology, Medical Faculty and University Hospital Duesseldorf, Heinrich Heine University Düsseldorf, Moorenstrasse 5, 40225 Duesseldorf, Germany; robert.bock@med.uni-duesseldorf.de (R.B.); maria.borrelli@med.uni-duesseldorf.de (M.B.)

There was an error in the original publication [1]. The supplementary material was listed as a software output instead of presenting the data in a human-readable Excel file.

A correction has been made to the Supplementary Material.

The authors state that the scientific conclusions are unaffected. This correction was approved by the Academic Editor. The original publication has also been updated.
